# Video-based feedback of oral clinical presentations reduces the anxiety of ICU medical students: a multicentre, prospective, randomized study

**DOI:** 10.1186/1472-6920-14-103

**Published:** 2014-05-22

**Authors:** Matthieu Schmidt, Yonathan Freund, Mickael Alves, Antoine Monsel, Vincent Labbe, Elsa Darnal, Jonathan Messika, Jerome Bokobza, Thomas Similowski, Alexandre Duguet

**Affiliations:** 1 Sorbonne Universités, UPMC Univ Paris 06, UMR_S 1158 “Neurophysiologie Respiratoire Expérimentale et Clinique”, Paris F-75005, France; 2 INSERM, UMR_S 1158 “Neurophysiologie Respiratoire Expérimentale et Clinique”, Paris F-75005, France; 3 AP-HP, Groupe Hospitalier Pitié-Salpêtrière Charles Foix, Service de Pneumologie et Réanimation Médicale (Département “R3S”), Paris F-75013, France; 4 Emergency Department, Hôpital Pitié-Salpêtrière and INSERM U956, Université Pierre et Marie Curie, Paris, France; 5 Department of Medical ICU, Hôpital Saint Antoine and Université Pierre et Marie Curie, Paris, France; 6 Department of Surgical ICU, Hôpital Pitié-Salpêtrière and Université Pierre et Marie Curie, Paris, France; 7 Department of Medical and Surgical ICU, Hôpital Tenon and Université Pierre et Marie Curie, Paris, France; 8 Emergency Department, Hôpital Pitié-Salpêtrière, Université Pierre et Marie Curie, Paris, France

**Keywords:** Oral feedback, Video tape, Medical education

## Abstract

**Background:**

Oral presentations of clinical cases by medical students during medical rounds in hospital wards are a source of anxiety and little is known about how this anxiety can be alleviated. The objective of this study was to investigate whether video-based feedback of public oral presentations can reduce anxiety in 4th year medical students.

**Methods:**

Multicentre randomized study conducted in six intensive care units (ICU) and emergency departments (ED) in France over a 9-month period in 2012. One hundred and forty two 4th year medical students were randomized to two groups: intervention and control. Students in the intervention group were recorded while making an oral presentation of a patient during morning ward rounds, followed by video-based feedback. Students in the control group conducted presented classical oral presentations without being filmed and with no formal feedback. Anxiety levels during a public oral presentation were assessed using the Spielberger State Anxiety Inventory (STAI-S). The primary outcome was the difference in STAI-S scores between groups at the beginning and at the end of a 3-month ICU or ED internship.

**Results:**

Seventy four students were randomized to the ‘video-based feedback’ group and 68 were randomized to the control group. In both groups, STAI-S scores were significantly lower after 3 months of internship. However, the reduction in STAI-S scores was significantly greater in the “video-based feedback” group than in controls (-9.2 ± 9.3 vs. –4.6 ± 8.2, *p* = 0.024. Compared to the control group, significantly fewer students with high-level anxiety were observed in the “video-based feedback” group after 3 months of internship (68 vs. 28%, *p* <0.001).

**Conclusions:**

Compared to “usual practice”, video-assisted oral feedback reduced anxiety and significantly decreased the proportion of students experiencing severe anxiety.

## Background

Oral presentations of clinical cases are performed daily by medical students during medical rounds in hospital wards. Public oral presentations are anxiogenic, particularly when they are associated with direct professional implications. However, the anxiety induced by oral presentations is alleviated only after years of experience, and little is known about how this anxiety can be reduced. Anxiety can have multiple consequences on the student’s personal and academic development [1-3]. Medical students [4,5] often report that anxiety increases their feelings of personal inadequacy [6]. Anxiety may also have a negative impact on the quality of the presentation, which may be a source of medical errors and which may affect the patient’s outcome [7-9]. It is therefore important to improve communication skills during medical training by ensuring a positive frame of mind.

Video-based feedback is already used in medicine, and good results [10-12] have been obtained in resuscitation of cardiac arrest [11,13] and surgical techniques using video-based feedback [14-16] have been shown to improve the efficacy of simulation-based teaching [17,18]. Similarly, the oral communication skills of psychiatrists are significantly improved after receiving feedback on their previous videotaped interviews [14-16,19]. Although there is undisputed evidence supporting the efficacy of video-based feedback when teaching clinical skills, the specific value of video-based feedback to reduce the anxiety of medical students has not been previously investigated. We hypothesized that systematic videotape-assisted feedback, as a composite teaching tool, decreases the anxiety of medical students. To test this hypothesis, we evaluated whether the students’ anxiety was reduced after receiving feedback from videotaped oral presentations compared with “usual” oral presentations without formal feedback. We also evaluated the students’ perceptions of this new educational tool.

## Methods

This multicentre, prospective, randomized, controlled study was conducted in six departments (three medical intensive care units (ICU), two surgical ICUs, and one emergency department (ED)) in urban university-affiliated hospitals (Université Paris 6 Pierre et Marie Curie), over a 9-month period in 2012. The protocol was approved by an independent institutional review board (“Comité de Protection des Personnes”, Paris Ile de France VI). All participants gave their written informed consent.

### Study population

After their first two years in medical school, French medical students spend half of their time in hospital and change wards 3 times a year. During this in-hospital training period, they learn how to examine patients, write medical reports, and apply their theoretical knowledge to various clinical settings. They also attend ward rounds with a senior physician and receive training in general medical practice.

At our university, 4^th^ year medical students must complete a mandatory 3-month training period in an ICU or ED. All students completing a 3-month internship in each participating department (i.e. three 3-month internships) were asked to participate in this study. Foreign-exchange students were not involved, as they follow a different curriculum.

### Study design

Eligible students were invited to participate in the study during the first 2 days of their ICU/ED training. Students were informed that the primary objective of the study was to test the impact of VBF on the level of anxiety induced by oral presentation. After being given a brief description of the study and after signing the informed consent form, medical students were asked to fill in an online questionnaire on sociodemographic data and anxiety assessment (see below). The students were then randomized on the same day using the Excel® (Excel 2007, Microsoft) random-number generation function. The medical students were assigned to either the “video-based feedback” (VBF) group or the control group (i.e. with no video feedback). In both arms, students had to perform a formal oral presentation of a patient on a regular basis during morning ward rounds or during handovers. Paper or clinical file supports were available for the student during the presentation. Depending on the group to which the student was randomized, the oral presentation was taped by means of a small portable camera on a mini-tripod (Q3 Handy Video Recorder, Zoom, Japan) or was not taped.

In the VBF group, after completing the ward rounds/handover, a senior physician performed formal feedback on the content and structure of the student’s oral presentation, in the presence of all other VBF students at the same centre, but with no students from the control group. Students of the control group were not filmed and did not receive any formal feedback, in line with standard practice in our wards: comments and criticisms of the oral presentation were left to the physician’s discretion in each centre. At the end of their training (i.e. 3 months), all students again filled in the same online questionnaire.

### Questionnaire content

Sociodemographic data were collected from all students at the beginning of their internship. Anxiety levels were assessed using the validated French version of the Spielberger State Anxiety Inventory (STAI-S) [20]. This score measures the transitional emotional status evoked by a stressful situation, such as an oral presentation, using 20 items each rated by a 4-point Likert scale (possible score range: 20–80). Higher scores are positively correlated with higher levels of anxiety. A score >37 for men and >42 for women reflects high anxiety, and a score >48 for men and >55 for women corresponds to anxiety liable to interfere with performance [21]. The level of anxiety was evaluated in all students at the beginning and at the end of the 3-month emergency room or ICU internship.

All online questionnaires were self-administered by the students. In addition to the STAI-S questionnaire, the students were asked for feedback on their perception and satisfaction with the filmed observation method. The first questionnaire was completed before randomization and on the first day of hospital training.

### Endpoints

The primary endpoint was a reduction in the STAI-S score. Secondary endpoints were: 1) the proportion of students with high anxiety and anxiety that might interfere with their oral presentation; and 2) the student’s perception of the “educational tool”.

### Statistical analysis

This study followed CONSORT recommendations for reporting randomized and controlled trials.

Sample size was calculated using the STAI-S value of typical healthy French students [20], which indicated that 141 students were needed to show a 5-point reduction in STAI-S score between the beginning and end of internship, with a power of 80% and a *P*-value of 0.05.

All data distributions were normal according to the Kolmogorov–Smirnov test. The data were therefore expressed as mean ± SD. Continuous variables were compared with Student’s t-test, whereas categorical variables were compared with a chi-square test. The primary endpoint (i.e. reduction in the STAI-S) in each group was compared using Student’s t-test. The proportion of students with high anxiety and anxiety that might interfere with the oral presentation at the beginning and at the end of the 3-month internship were compared using the paired McNemar test.

All *P* values were two-tailed and *P* <0.05 was considered significant. Statistical analyses were performed using StatView 5.0 (SAS Institute Inc., Cary, NC) software.

## Results

A total of 150 4^th^ year medical students were enrolled in the study over a 9-month period. Two students refused to participate, while 8 students were excluded from the analysis due to insufficient data. Seventy four students were randomized -to the ‘video-based feedback’ group and 68 four students were randomized to the control group.

### Population characteristics

The characteristics and mean pre-intervention STAI-S scores for each group are summarized in Table 1. No significant differences were observed between the two groups. Of note, 44% of all students reported being shy, and 38% reported being anxious during their hospital training. In addition, before randomization, 55% (*n* = 79) of students reported being afraid of speaking in public. Each of the 74 students in the VBF group received a mean of 6 ± 1 VBF sessions devoted to their own performance during the 3-month period: each lasted 16 ± 7 min, while students in the control group did not receive any formal feedback.

**Table 1:** Student characteristics and pre-randomisation perceptions of anxiety and public speaking

	**Total (*n*** **= 142)**	**Control group (*n*** **= 68)**	**Video-based feedback group (*n*** **= 74)**	***P***
Age, years	22 ± 1	22 ± 1	22 ± 1	0.61
Male, *n* (%)	45 (32)	24 (35)	21 (29)	0.37
Students’ perceptions before randomization, *n* (%):				
“I am shy at hospital”	63 (44)	32 (51)	31 (49)	0.38
“I am anxious during hospital internship”	54 (38)	28 (41)	26 (35)	0.54
“I am afraid to speak in public”	79 (55)	36 (53)	43 (58)	0.38
“I am anxious to be filmed”	99 (70)	49 (72)	50 (67)	0.56
“I fear the criticism of doctors from my department”	93 (65)	41 (60)	52 (70)	0.21
“I fear the criticism of other students”	75 (53)	34 (50)	41 (55)	0.51
Spielberger State Anxiety Inventory (STAI-S) score during an oral presentation before randomization^$^	44 ± 9	42 ± 9	45 ± 9	0.10
- High anxiety*	82 (58)	36 (53)	46 (62)	0.26
- Major anxiety that could interfere with the student’s performance**	24 (17)	9 (13)	15 (38)	0.26

^$^Spielberger State Anxiety Inventory (STAI-S), score from 20–80.

*Significantly elevated anxiety is defined as a STAI-S score of >37 for men and >42 for women.

**Anxiety that could interfere with the student’s performance was defined as a STAI-S score of >48 for men and >55 for women.

Data are expressed as *n* (%) or mean ± SD.

### Self-assessment of anxiety generated by an oral presentation

Fifty-eight per cent (*n* = 82) of students reported significant anxiety (i.e., STAI-S score >37 for men and >42 for women) during oral presentations at the beginning of their ER or ICU internship: 62% in the VBF group vs. 53% in the control group (*p* = 0.26). In addition, 17% reported that this high level of anxiety interfered with the quality of their oral presentation (Table 1).

### Impact of video-based feedback

**Figure 1:**
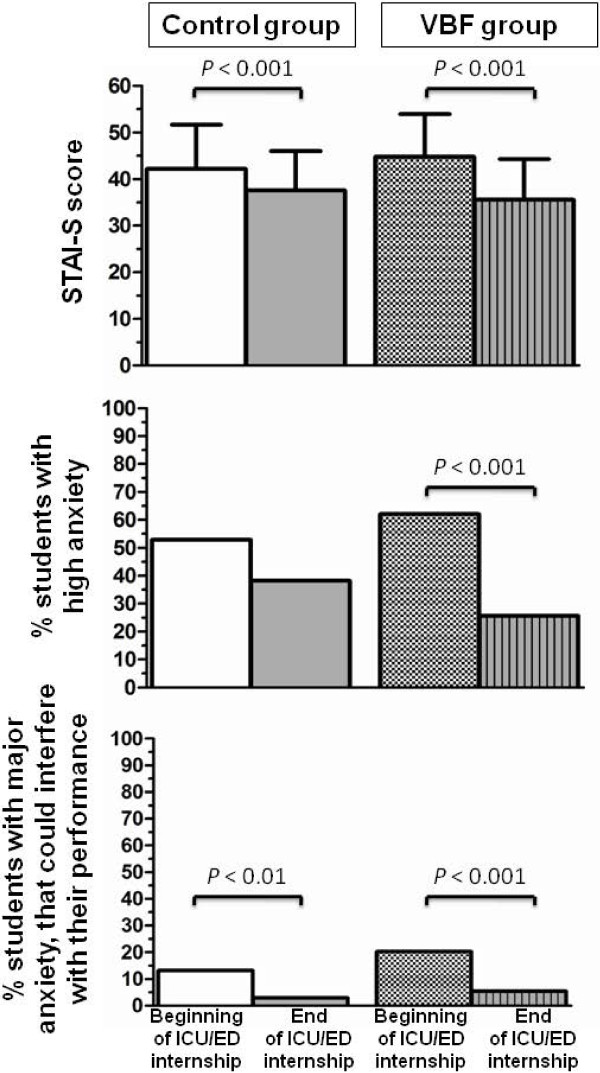
**Self-assessment of anxiety generated by oral presentation at the beginning and end of an intensive care unit or emergency department internship.** *STAI-S*, Spielberger State Anxiety Inventory: scores from 20–80. The *VBF* group, with video-based feedback; the *control group*, with no video-based feedback; *ICU,* intensive care unit; *ED,* emergency department.

The STAI-S scores and the numbers of students with major anxiety that could interfere with their performance significantly decreased in both groups by the end of their internship. However, the reduction in the STAI-S score was significantly greater in the VBF group compared to the control group (-9.2 ± 9.3 vs. –4.6 ± 8.2, *p* = 0.024). Similarly, the number of students with high anxiety was markedly and significantly lower in the VBF group than in the control group after 3 months of internship (62 vs. 28%, *p* <0.001) (Figure 1).

### Students’ perceptions of VBF

Video recording of oral presentations was described as a stressful experience, but, overall, most students in the VBF group reported a favourable memory of the experience (Table 2). Seventy-seven percent of students in the video group reported that this “teaching experience” should be extended to internships in other wards. Similarly, 74% of students in the control group regretted not having received personalized debriefing of their oral presentations. Only two students refused to take part in this study, reflecting the good adherence of the students.

**Table 2:** Students’ perceptions of video-based feedback

	**No**	**Rather no**	**Rather yes**	**Yes**
**Video-based feedback (VBF) group (*n*** **= 74)**				
“I have a fond memory of the internship”	1 (1)	10 (13)	23 (30)	43 (56)
“This was a weak point of the internship”	65 (84)	11 (14)	1 (2)	0 (0)
“This “experience” has helped me to “generalize”	7 (9)	11 (14)	23 (30)	36 (47)
“This was a stressful time of the internship”	24 (31)	19 (25)	28 (36)	6 (8)
“I’m glad it is over”	18 (23)	32 (41)	21 (27)	6 (8)
**No video-based feedback (nVBF) group (*n*** **= 68)**				
“I’m disappointed that I wasn’t filmed”	18 (28)	16 (25)	16 (25)	14 (22)
“I’m disappointed that I was not debriefed”	12 (19)	4 (6)	15 (23)	33 (51)
“My oral presentations could have been improved by video-assisted feedback”	9 (14)	3 (5)	30 (47)	22 (34)

## Discussion

This study confirms that oral presentation is a major source of anxiety for medical students. Video-based feedback significantly amplified the anxiety-attenuating effects of repeating public oral presentations and the associated “oral” feedback during a 3-month internship period. It also decreased the proportion of students with anxiety sufficiently severe to impair their performance.

To our knowledge, this is the first study to evaluate the benefit of VBF on anxiety generated by oral presentation during ward rounds. The principle of debriefing is a classical element in project management, sport training, and, more recently, in simulation-based medical teaching [22,23]. Debriefing can focus on positive aspects, can identify failures, and can suggest corrective actions to remedy mistakes made during the presentation. The immediate effect of debriefing immediately after the oral presentation in the presence of other students who can make constructive criticisms is to reduce the accumulated pressure and stress experienced by the student who is being appraised. However, the methods used to conduct feedback are of utmost importance. Empathy during video debriefing is more effective that harsh criticism to avoid demotivating the student and decreasing his/her future performance [5,24]. The students in our study showed good adhesion to these debriefings, as suggested by the significant decrease in anxiety after VBF. The students were also keen to extend this concept to subsequent training sessions (Table 2).

Feedback now appears to be an essential part of medical simulations and education [22,25,26]. Some authors suggest that the addition of video review does not provide any advantages over oral feedback alone [22,27]. However, we believe that VBF increases the didactic impact of the feedback [28,29]. Previous studies in the field of medical education demonstrate that VBF improves efficiency when participants have several opportunities to review their performance [28,29]. Repeated and targeted VBF (mean: 6 ± 1 times) in our study may therefore have contributed to significantly reducing the anxiety of students in the VBF group.

Public oral presentation is difficult and causes high levels of distress to many students. The STAI-S score before randomization was 41 ± 9 for men and 45 ± 9 for women, respectively. In comparison, similar STAI-S scores were found in a French population of patients with burn injuries (42 ± 12 and 45 ± 10 for men and women, respectively) or before a surgical operation (41 ± 9 and 45 ± 8) [20]. In addition, 58% of our students experienced high anxiety levels during an oral presentation at the beginning of their internship, and 17% experience anxiety that was so severe as to interfere with their performance (Table 1).

Just as a coach teaches athletes how to cope with stress before a competition, senior physicians should try to decrease the anxiety induced by oral presentations. A VBF could help achieve this goal (Figure 1). To the best of our knowledge, no formal training is available to help medical students with oral presentations. We consider this anxiety to be a matter for concern. VBF also generates a positive dynamic within the debriefed group and reduces inter-student resentments [3,30]. Lastly, because anxiety can interfere with performance [31-33], VBF may also have enhanced the quality of oral presentations.

This study has several limitations. Firstly, this study was not designed to demonstrate a specific benefit of the videotaped presentation alone. The study was designed to assess the combined effect of videotaping, formal debriefing and feedback, rather than the sole added value of videotaping. In order to address the specific benefits of video to reduce anxiety, a future study would need to compare formal versus video-assisted oral case presentations, with similar debriefing and feedback in both groups. Secondly, feedback in the control group was not standardized. Because the study was probably the subject of many informal discussions between students, it is possible that even those in the control group received some advice from their fellow students. This possible crossover could partially explain the significant decrease in the STAI-S score after the 3-month period, even in the control group. Thirdly, the higher baseline STAI-S score in the VBF group, although not significant, could partially explain the more marked reduction of the STAI-S score at the end of the study. Lastly, the Hawthorne effect [34], a situation in which the results of an experiment are not caused by experimental factors, but rather because the subjects were aware that they were tested, is an inherent limitation to this type of study and cannot be eliminated.

## Conclusions

Oral case presentations by medical students are part of the daily routine in ICUs and ERs, though they can often be stressful. Video-assisted review of oral presentations is simple, not time-consuming, and is very popular. Investment in this educational methodology could reduce major anxiety after only a short period. However, the specific impact of feedback on the quality of the oral presentation needs to be investigated in future studies.

## Abbreviations

ED: Emergency department; ICU: Intensive-care units; STAI-S: Spielberger State Anxiety Inventory; VBF: Video-based feedback.

## Competing interests

The authors declare that they have no competing interests.

## Authors’ contributions

Conception and design: MS, YF, TS, AD. Acquisition of data: MS, YF, MA, AM, VL, ED, JM, JB. Analysis and interpretation of data: MS, YF, TS, AD. Drafting the article: MS, YF, TS, AD. Revising it critically for important intellectual content: MA, AM, VL, ED, JM, JB, TS, AD. Final approval of the version to be published: MS, YF, MA, AM, VL, ED, JM, JB, TS, AD.

## Authors’ information

Dr Schmidt Matthieu is a fellow in the Department of Pneumology and Medical ICU, Hôpital Pitié-Salpêtrière and Université Pierre et Marie Curie, Paris, France.

Dr Freund Yonathan is a fellow in the Emergency Department, Hôpital Pitié-Salpêtrière and INSERM U956, Université Pierre et Marie Curie, Paris, France.

Dr Alves Mickael is a fellow in the Department of Medical ICU, Hôpital Saint Antoine and Université Pierre et Marie Curie, Paris, France.

Dr Labbe Vincent and Dr Messika Jonathan are fellows in the Department of Medical and Surgical ICU, Hôpital Tenon and Université Pierre et Marie Curie, Paris, France.

Dr Monsel Antoine and Dr Darnal Elsa are fellows in the Department of Surgical ICU, Hôpital Pitié-Salpêtrière and Université Pierre et Marie Curie, Paris, France.

Dr Bokobza Jerome is a fellow in the Emergency Department, Hôpital Pitié-Salpêtrière, Université Pierre et Marie Curie, Paris, France.

Prof. Similowski Thomas is professor and Chairman of the Department of Pneumology and Medical ICU, Hôpital Pitié-Salpêtrière and Université Pierre et Marie Curie, Paris, France.

Prof. Duguet Alexandre is professor in the Department of Pneumology and Medical ICU, Hôpital Pitié-Salpêtrière and Université Pierre et Marie Curie, Paris, France.

## Pre-publication history

The pre-publication history for this paper can be accessed here:

http://www.biomedcentral.com/1472-6920/14/103/prepub

## References

[B1] Spiegel DA Smolen RC Hopfensperger KA Medical student stress and clerkship performance J Med Educ 1986 61 929 931 3772973 10.1097/00001888-198611000-00015

[B2] Spiegel DA Smolen RC Jonas CK An examination of the relationships among interpersonal stress, morale and academic performance in male and female medical students Soc Sci Med 1986 23 1157 1161 10.1016/0277-9536(86)90334-5 3810201

[B3] Dyrbye LN Thomas MR Shanafelt TD Medical student distress: causes, consequences, and proposed solutions Mayo Clin Proc 2005 80 1613 1622 10.4065/80.12.1613 16342655

[B4] Wolf TM Randall HM von Almen K Tynes LL Perceived mistreatment and attitude change by graduating medical students: a retrospective study Med Educ 1991 25 182 190 10.1111/j.1365-2923.1991.tb00050.x 1857273

[B5] Wilkinson TJ Gill DJ Fitzjohn J Palmer CL Mulder RT The impact on students of adverse experiences during medical school Med Teach 2006 28 129 135 10.1080/01421590600607195 16707293

[B6] Benbassat J Undesirable features of the medical learning environment: a narrative review of the literature Adv Health Sci Educ Theory Pract 2013 18 3 527 536 10.1007/s10459-012-9389-5 22760724

[B7] Leonard M Graham S Bonacum D The human factor: the critical importance of effective teamwork and communication in providing safe care Qual Saf Health Care 2004 13 Suppl 1 i85 i90 15465961 10.1136/qshc.2004.010033 PMC1765783

[B8] Lingard L Espin S Whyte S Regehr G Baker GR Reznick R Bohnen J Orser B Doran D Grober E Communication failures in the operating room: an observational classification of recurrent types and effects Qual Saf Health Care 2004 13 330 334 10.1136/qshc.2003.008425 15465935 PMC1743897

[B9] Williams RG Silverman R Schwind C Fortune JB Sutyak J Horvath KD Van Eaton EG Azzie G Potts JR 3rd Boehler M Dunnington GL Surgeon information transfer and communication: factors affecting quality and efficiency of inpatient care Ann Surg 2007 245 159 169 10.1097/01.sla.0000242709.28760.56 17245166 PMC1877003

[B10] Lockyer J Armson H Chesluk B Dornan T Holmboe E Loney E Mann K Sargeant J Feedback data sources that inform physician self-assessment Med Teach 2011 33 e113 e120 10.3109/0142159X.2011.542519 21275533

[B11] Allan CK Thiagarajan RR Beke D Imprescia A Kappus LJ Garden A Hayes G Laussen PC Bacha E Weinstock PH Simulation-based training delivered directly to the pediatric cardiac intensive care unit engenders preparedness, comfort, and decreased anxiety among multidisciplinary resuscitation teams J Thorac Cardiovasc Surg 2010 140 646 652 10.1016/j.jtcvs.2010.04.027 20570292

[B12] Howe A Detecting psychological distress: can general practitioners improve their own performance? Br J Gen Pract 1996 46 407 410 8776911 PMC1239692

[B13] Li Q Ma E-L Liu J Fang L-Q Xia T Pre-training evaluation and feedback improve medical students’ skills in basic life support Med Teach 2011 33 e549 e555 10.3109/0142159X.2011.600360 21942491

[B14] Snyder CW Vandromme MJ Tyra SL Porterfield JR Jr Clements RH Hawn MT Effects of virtual reality simulator training method and observational learning on surgical performance World J Surg 2011 35 245 252 10.1007/s00268-010-0861-1 21086125

[B15] Solomon B Bizekis C Dellis SL Donington JS Oliker A Balsam LB Zervos M Galloway AC Pass H Grossi EA Simulating video-assisted thoracoscopic lobectomy: a virtual reality cognitive task simulation J Thorac Cardiovasc Surg 2011 141 249 255 10.1016/j.jtcvs.2010.09.014 21168026

[B16] Joyce DL Dhillon TS Caffarelli AD Joyce DD Tsirigotis DN Burdon TA Fann JI Simulation and skills training in mitral valve surgery J Thorac Cardiovasc Surg 2011 141 107 112 10.1016/j.jtcvs.2010.08.059 21074189

[B17] Hamilton NA Kieninger AN Woodhouse J Freeman BD Murray D Klingensmith ME Video review using a reliable evaluation metric improves team function in high-fidelity simulated trauma resuscitation J Surg Educ 2012 69 428 431 10.1016/j.jsurg.2011.09.009 22483149

[B18] Owen H Sprick C Simulation debriefing and quantitative analysis using video analysis software Stud Health Technol Inform 2008 132 345 347 18391318

[B19] Gask L Goldberg D Lesser AL Millar T Improving the psychiatric skills of the general practice trainee: an evaluation of a group training course Med Educ 1988 22 132 138 10.1111/j.1365-2923.1988.tb00423.x 3374414

[B20] Spielberger C Gorsuch R Lushene R Vagg P Jacobs G Manuel de L’inventaire D’anxiété État-Trait Forme Y (STAI-Y). Editions du Centre de Psychologie Appliquée 1993 Paris: (M. Bruchon-Schweitzer & I. Paulhan, Adapt. Française)

[B21] Spielberger C Manual for the State-Trait Anxiety Inventory 1983 Palo Alto, California: Consulting Psychologists Press

[B22] Savoldelli GL Naik VN Park J Joo HS Chow R Hamstra SJ Value of debriefing during simulated crisis management: oral versus video-assisted oral feedback Anesthesiology 2006 105 279 285 10.1097/00000542-200608000-00010 16871061

[B23] Fanning RM Gaba DM The role of debriefing in simulation-based learning Simul Healthc 2007 2 115 125 10.1097/SIH.0b013e3180315539 19088616

[B24] Lempp H Seale C The hidden curriculum in undergraduate medical education: qualitative study of medical students’ perceptions of teaching BMJ 2004 329 770 773 10.1136/bmj.329.7469.770 15459051 PMC520997

[B25] Morgan PJ Kurrek MM Bertram S LeBlanc V Przybyszewski T Nontechnical skills assessment after simulation-based continuing medical education Simul Healthc 2011 6 255 259 10.1097/SIH.0b013e31821dfd05 21642904

[B26] Hart D McNeil MA Griswold-Theodorson S Bhatia K Joing S High fidelity case-based simulation debriefing: everything you need to know Acad Emerg Med 2012 19 E1084 10.1111/j.1553-2712.2012.01423.x 22978737

[B27] Hecimovich MD Maire J-A Losco B Effect of Clinician Feedback Versus Video Self-Assessment in 5th-Year Chiropractic Students on an End-of-Year Communication Skills Examination J Chiropr Educ 2010 24 165 174 10.7899/1042-5055-24.2.165 21048879 PMC2967341

[B28] Birnbach DJ Santos AC Bourlier RA Meadows WE Datta S Stein DJ Kuroda MM Thys DM The effectiveness of video technology as an adjunct to teach and evaluate epidural anesthesia performance skills Anesthesiology 2002 96 5 9 10.1097/00000542-200201000-00007 11752994

[B29] Scherer LA Chang MC Meredith JW Battistella FD Videotape review leads to rapid and sustained learning Am J Surg 2003 185 516 520 10.1016/S0002-9610(03)00062-X 12781877

[B30] Sheehan KH Sheehan DV White K Leibowitz A Baldwin DC Jr A pilot study of medical student “abuse”. Student perceptions of mistreatment and misconduct in medical school JAMA 1990 263 533 537 10.1001/jama.1990.03440040072031 2294325

[B31] Vytal K Cornwell B Arkin N Grillon C Describing the interplay between anxiety and cognition: from impaired performance under low cognitive load to reduced anxiety under high load Psychophysiology 2012 49 842 852 10.1111/j.1469-8986.2012.01358.x 22332819 PMC3345059

[B32] Choi JM Padmala S Pessoa L Impact of state anxiety on the interaction between threat monitoring and cognition Neuroimage 2012 59 1912 1923 10.1016/j.neuroimage.2011.08.102 21939773 PMC3230669

[B33] Tennyson RD Woolley FR Interaction of anxiety with performance on two levels of task difficulty J Educ Psychol 1971 62 463 467 5130019 10.1037/h0031821

[B34] Hsueh Y The Hawthorne experiments and the introduction of Jean Piaget in American industrial psychology, 1929–1932 Hist Psychol 2002 5 163 189 12096759 10.1037/1093-4510.5.2.163

